# Mass screening for colorectal cancer in a population of two million older adults in Guangzhou, China

**DOI:** 10.1038/s41598-019-46670-2

**Published:** 2019-07-18

**Authors:** Guozhen Lin, Zhiqiang Feng, Huazhang Liu, Yan Li, Yuqiang Nie, Yingru Liang, Ke Li

**Affiliations:** 1 Colorectal Cancer Screening Programme, Guangzhou Center for Disease Control and Prevention, Guangzhou, 510440 China; 2 Department of Gastroenterology, Guangzhou First People’s Hospital, School of Medicine, South China University of Technology, Guangzhou, 510180 China; 3 Cancer Registry, Guangzhou Center for Disease Control and Prevention, Guangzhou, 510440 China

**Keywords:** Cancer screening, Colorectal cancer

## Abstract

Screening is an effective measure to prevent and control colorectal cancer (CRC). A mass CRC screening programme was conducted in Guangzhou from 2015 to 2017. Public media and reminders from a mobile short message service were used to invite residents aged between 50 and 74 years. A high-risk factor questionnaire (HRFQ) and biennial faecal immunochemical testing (FIT) were chosen as the primary screening methods, and individuals with a positive test result were referred to a defined hospital for an assessment colonoscopy. During the 3 years, 350,581 residents of the total eligible population of 2,283,214 attended the free first stage of screening. In all, 91.0% of the participants finished the HRFQs and FITs. The total uptake rate was 15.4%, which increased with age, female sex, and rural location. There was 15.9% positivity in the first stage of screening, including 8.5% positive HRFQs, 6.2% positive FITs and 1.2% positive HRFQs and FITs. In total, 10,600 individuals with positive HRFQs/FITs completed an assessment colonoscopy. The total uptake rate of colonoscopies was 18.9%, which decreased with age and female sex. Three hundred fifty-one CRCs and 980 advanced adenomas (AAs) were diagnosed with positive predictive values (PPV) of 3.3% and 9.2%, respectively. The PPVs of CRCs in the exclusively FIT-positive population were 4.9%, which was 10 times greater than in the exclusively HRFQ-positive population (0.5%). The PPVs of CRCs and AAs increased with age and male sex. The detection ratio of localized CRCs (including stage I and stage II) increased 68.1% due to screening. Although the compliance rate was low, the PPVs for CRCs and AAs were high. More effective mobilization of the programme’s needs and subsidies for colonoscopies should be taken into account to increase compliance.

## Introduction

Colorectal cancer (CRC) is the third most common malignancy in men and the second most common in women, with approximately 1,360,000 newly diagnosed cases globally and approximately 694,000 deaths in 2012, and it is one of the leading causes of global morbidity and mortality^1^. With accelerating urbanization and changing lifestyles in recent decades, many countries in Asia, especially China, have been experiencing a rapid rise in the incidence of CRC^2^. The incidence of colorectal cancer has ranked first in Taiwan since 2009^3^ and in Hong Kong since 2012^4^. In Guangzhou, the third largest city in mainland China with a population of over 8 million in 2017, the incidence of colorectal cancer is still moderate with a morbidity of 34/100,000 and a mortality of 17/100,000 in 2011, while the trend of the incidence rate is obviously increasing over time^5^.

Screening can identify and thus lead to the cure of more patients with early-stage colorectal cancer. Moreover, most colorectal cancers develop from colorectal adenomas (known as precursor lesions), and the process usually takes up to 10 years^6,7^. Most colorectal adenomas can be detected by colonoscopy, and there are effective treatments. The early detection and removal of colorectal adenomas has proven to be an efficient means of reducing the incidence of colorectal cancer^8,9^. There are several different screening strategies. Adenomas can be removed during colonoscopy screening, and the procedure is cost-effective. Therefore, colonoscopy is recommended as the first choice for the public in many countries^10^. However, the low uptake of colonoscopy is still an obstacle for researchers to overcome^11^. Many studies have demonstrated that guaiac faecal occult blood tests (FOBT) are a cost-effective CRC screening method^12,13^, while faecal immunochemical testing (FIT) has garnered more attention and has gained higher sensitivity in recent years^14^. In addition, Zheng *et al*.^15^ established a high-risk factor questionnaire as a screening tool for colorectal cancer after a series of epidemiologic risk factor studies in the Chinese population. This score, combined with the FOBT, followed by colonoscopy if either the questionnaire or test is positive, has been proven to be effective^16^ and has been accepted as the protocol for colorectal screening by the China National Committee of Cancer Early Detection and Treatment^17^. The City Authority of Guangzhou decided to introduce the first round of mass CRC screening from 2015 to 2017, according to local expert advice after many hearings and discussions. There are increasing numbers of screenings beginning in different countries and areas, and pioneer studies have been implemented in China^18–20^. We evaluated the compliance and yield by age, sex, and residence to provide useful references for researchers.

## Methods

### Study design

Demographic data of all individuals between 50 and 74 years living in Guangzhou were obtained from the municipal population registry. In February 2015, a conference was held by the Guangzhou Health Department to announce the colorectal cancer screening programme. Newspapers and TVs reported that the Guangzhou government invited local residents to the screening. Every local resident aged 50 to 74 years received SMS reminders. Individuals who had previous CRCs; had undergone a colonoscopy, sigmoidoscopy, or barium contrast enema in the past 5 years; or who did not give informed consent were excluded from the study. A two-stage screening was used in accordance with the recommended guideline^17^. FITs and HRFQs were used as the primary screening methods in the first stage. FITs (test kits produced by W.H.P.M., Inc. Beijing, China) were repeated once after 1 week. The test result was considered positive when the haemoglobin concentration in at least one sample was ≥100 ng/ml, which corresponds to ≥20 μg Hb/g faeces. The HRFQ included basic demographic information, such as age, sex, residence, marital status, and education level, and 9 CRC risk factor questions. A positive HRFQ means:

(i) Individuals had one of the following events: (a) a history of cancer, (b) a history of polyps, or (c) a family history of CRC in a first-degree relative; and/or
(ii) At least 2 of the following events: (a) chronic coprostasis; (b) chronic diarrhoea; (c) mucoid bloody faeces; (d) serious unhappy life events, such as the death of a first-degree relative; (e) chronic appendicitis or appendectomy; or (f) chronic cholecystitis or cholecystectomy^15,17^.

**Figure 1:**
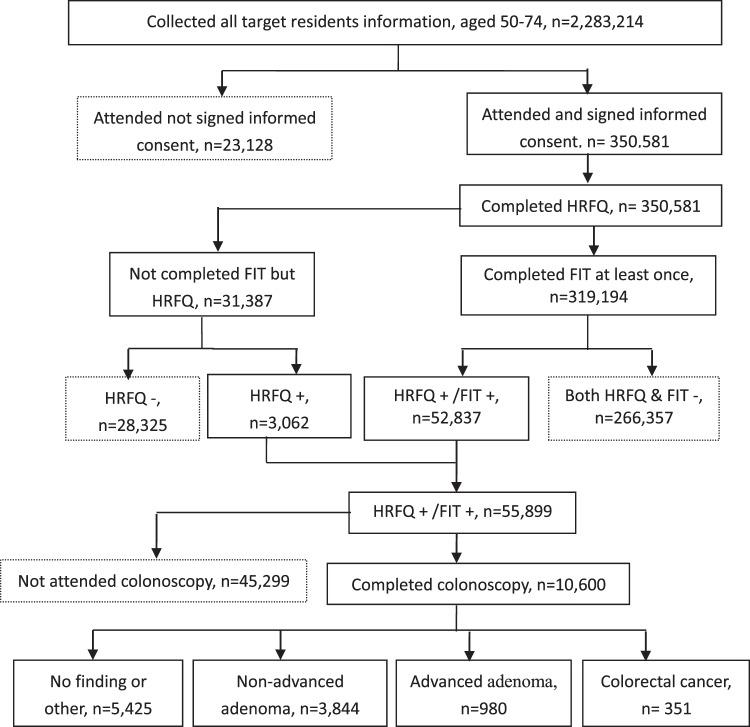
Flow chart of colorectal cancer screening in Guangzhou, 2015–2017.

The administration of the HRFQ was completed by trained general practitioners (GPs) in local community health centres. Faecal samples were collected by community health workers and tested in the same health centres by experienced technicians. The municipal government provided subsidies for this project to cover the HRFQs and FITs. If either the FIT or HRFQ was positive, the patient was recommended to undergo a colonoscopy by a GP and transferred to designated hospitals with the capacities to perform colonoscopies and CRC multidisciplinary teams in the second stage. Experienced endoscopists who were certified and had performed over 1,000 colonoscopies performed all screening colonoscopies. The endoscopists recorded the maximum reach of the endoscope, adequacy of bowel preparation, and the characteristics and locations of any adenomas. Patients with positive findings on colonoscopy entered a surveillance programme according to guidelines of the Chinese Society of Digestive Endoscopy^17^. If a colonoscopy examination failed due to inadequate bowel preparation or the colonoscopy could not reach the caecum, a second colonoscopy was carried out within 1 month. All cancer cases (including deaths and new diagnoses) were enrolled in the Guangzhou Cancer Registration System. A flowchart of the CRC screening is presented in Fig. 1.

### Histopathologic examination

If a positive result was found on colonoscopy, a biopsy was performed, and gastrointestinal pathologists evaluated all removed polyps. On the basis of the International Classification, CRC was defined as the invasion of malignant cells beyond the muscular mucosa. Patients with intramucosal carcinoma or carcinoma *in situ* were classified as having high-grade dysplasia. Advanced adenoma was defined as an adenoma with a diameter ≥10 mm and/or with a ≥25% villous component and/or high-grade dysplasia. When more than one lesion was present, the participant was classified according to the most advanced lesion^17^. The histologic classification of all polyps included adenoma and non-adenomatous polyps (tubular, tubulovillous, or villous). Pathologic slides of positive lesions were re-examined and diagnosed by at least 2 independent pathologists.

### Follow-up after colonoscopy

The GPs in local community health centres followed every participant who completed a colonoscopy to collect data on any comorbidities related to the endoscopic procedure and to confirm the final diagnosis. Further examination and treatment were performed if CRC was reported. The chief doctor staged the patients with CRCs by synthesizing the results of biopsy, operation and imaging in accordance with the TNM staging system (8th edition) of the American Joint Committee on Cancer (AJCC)^21^.

### Data source

A specialized online system for CRC screening was established. There were data ports designed for community health centres and hospitals. The primary individual screening results of the HRFQs and FITs were entered into the computer by the GPs in local community health centres who performed the tests. Colonoscopy and histopathology results were recorded and entered into computers by endoscopists. The data were double checked to ensure quality after the final file was created. Guangzhou established an integrated cancer registration system in 1998 that covers the entire population of the city. In 2010, the registry successfully shifted to reporting cancer cases and follow-up of patients online^22^, and the data on incidence and mortality in Guangzhou has been accepted by the International Agency for Cancer Research (IARC) as content for Cancer Incidence in Five Continents (CI5) vol. XI^23^. To evaluate the early detection rates of CRC by screening, we chose 37 hospitals in which the screening colonoscopies were performed to collect all cases of colorectal cancer reported by the system from 2015 to 2017. Then, we deleted duplicate cases that existed in the screening group by comparing patient names, identification numbers and places of residence. The remaining cases were compared with cases found by screening.

### Statistical analysis

We calculated the uptake rate and the positivity rate for the first stage of the screening, and the positive predictive values (PPVs) for CRCs and AAs were calculated in the second stage colonoscopies. The uptake rate was calculated by dividing the number of participants by all eligible subjects (defined as all invitees minus the excluded subjects). The positivity rate was defined as the proportion of participants with a positive test result. The PPV referred to the proportion of subjects diagnosed with CRC/AA from among the participants with a positive HRFQ/FIT undergoing a subsequent colonoscopy. Differences in the proportions between groups were analysed by the χ^2^ test. The attendance rate, positivity rate and PPV were calculated and are described as proportions with 95% confidence intervals (95% CI). To calculate the increased detection ratio of localized CRC by screening, the result of the localized CRC detection ratio from screening minus the detection ratio from the system report was divided by the detection ratio of the system report. All P values were two-sided and considered significant if they were <0.05. All tests were conducted using SPSS version 20.0 for Windows (SPSS, Armonk, NY).

### Ethics approval

All the procedures were performed in accordance with the Declaration of Helsinki and relevant policies in China. The Guangzhou Health Council and the Review Board of the Guangzhou Center for Disease Control & Prevention approved the study (GZCDC2014006). All participants gave written informed consent.

## Results

### Attendance rate

According to the demographic data from the Statistics Bureau of Guangzhou, there were 2,283,214 individuals between 50 and 74 years old among the 8,399,378 local citizens in 2014. Although the population increases by approximately 9% every year because of immigration, the proportion of 50- to 74-year-old residents is stable. From 2015 to 2017, there were 350,581 residents who attended the free first stage of screening among the 2,283,214 eligible subjects in Guangzhou. In all, 91.0% (319,194/350,581) of attendants finished the HRFQs and FITs (once or twice). The total attendance rate was 15.4%, which increased with increasing age (χ^2^ = 55,097.420, p < 0.001). Comparing the attendance rate for the 65- to 69-year-old group to the attendance rate for the 70- to 74-year-old group, the former was higher at 27.3%, compared with 21.7% in the latter. The attendance rate of females was significantly higher than that of males (19.2% vs 11.5%, χ^2^ = 18,761.766, p < 0.001) and slightly higher in rural than in urban areas (15.9% vs 15.1%, χ^2^ = 221.411, p < 0.001). The total attendance rate for the FIT was only 14.0% (91% of 15.4%). In the second stage of screening, 10,600 participants completed the colonoscopies among the 55,899 positive residents in the population. The total attendance rate was 18.9%, which decreased with increasing age (χ^2^ = 457.348, p < 0.001). The attendance rate of females was lower than that of males (18.1% VS 20.3%, χ^2^ = 43.823, p < 0.001) and not different between rural and urban areas (χ^2^ = 0.193, p = 0.609) (Table 1).

**Table 1:** Overview of participation by age, gender and residence.

	Eligible invitees n	Participant^#^ n (%)	Positivity (%)				Colonoscopies performed (%) n (%)
			Total n (%)	HRFQ n (%)	FIT n (%)	Both n (%)	
**Age**, **years**							
Total	2283214	350581 (15.4)	55899 (15.9)	29798 (8.5)	21808 (6.2)	4295 (1.2)	10600 (18.9)
50–54	680321	57396 (8.4)	7817 (13.6)	4587 (8.0)	2663 (4.6)	567 (1.0)	1835 (23.5)
55–59	497790	58778 (11.8)	9299 (15.8)	5316 (9.0)	3110 (5.6)	673 (1.1)	1980 (21.3)
60–64	512085	85489 (16.7)	14025 (16.4)	7646 (8.9)	5299 (6.2)	1081 (1.3)	2988 (21.3)
65–69	361998	98832(27.3)	16262 (16.5)	8101 (8.2)	6894 (7.0)	1268 (1.3)	2719 (16.7)
70–74	231020	50086 (21.7)	8496 (17.0)	4148 (8.3)	3642 (7.3)	706 (1.4)	1078 (12.7)
χ^2^		55097.419	303.661	609.751			457.348
*P*		<0.001	<0.001	<0.001			<0.001
**Gender**							
Male	1132403	130361 (11.5)	22044 (16.9)	10859 (8.3)	9329 (7.2)	1859 (1.4)	4480 (20.3)
Female	1150811	220220 (19.2)	33855 (15.4)	18939 (9.7)	14916(7.4)	2437 (1.1)	6120 (18.1)
χ^2^		18761.766	144.283	390.076			43.823
*P*		<0.001	<0.001	<0.001			<0.001
**Residence**							
Urban	1500927	225967 (15.1)	42096 (18.6)	23523 (10.4)	15161 (6.7)	3414 (1.5)	8004 (19.0)
Rural	782287	124614 (15.9)	13803 (11.1)	6275 (5.0)	6647 (5.3)	881 (0.7)	2596 (18.8)
χ^2^		221.411	3418.558	3942.576			0.193
*P*		<0.001	<0.001	<0.001			0.609

^#^All participants finished HRFQ and 319194 invitees took part in FIT at least for one time. HRFQ: high-risk factor questionnaire, FIT: fecal immunochemical test.

### Proportion of positivity

In the first stage of screening, 15.9% (55,899/350,581) of subjects were positive, including 8.5% positive on the HRFQs, 6.2% positive on the FITs and 1.2% positive on both (Table 1). According to the trend chi square tests, the total positive rates (any one test of HRFQs and FITs) increased with increasing age (χ^2^ = 303.661, p < 0.001). When the positive population was divided into three subgroups, the positive rates increased sharply with increasing age (χ^2^ = 609.751, p < 0.001) in the positive FIT group. Male and urban subjects had higher positive rates than female subjects (16.9% vs 15.4%, χ^2^ = 144.283, p < 0.001) and rural subjects (18.6% vs 11.1%, χ^2^ = 3,418.558, p < 0.001). Males and females had similarly high rates of positivity (9.8% VS 9.7%, χ^2^ = 0.465, p = 0.497) on the HRFQs, while individuals from urban areas had significantly higher rates of positivity compared to those from rural areas (12.1% VS 5.7%, χ^2^ = 3,514.127, p < 0.001) on the HRFQs. Male and urban subjects had higher positive rates than female subjects (9.5% vs 6.3%, χ^2^ = 425.335, p < 0.001) and rural subjects (9.3% vs 6.3%, χ^2^ = 851.320, p < 0.001) on the FITs.

### Proportions of adenoma and cancer

**Figure 2:**
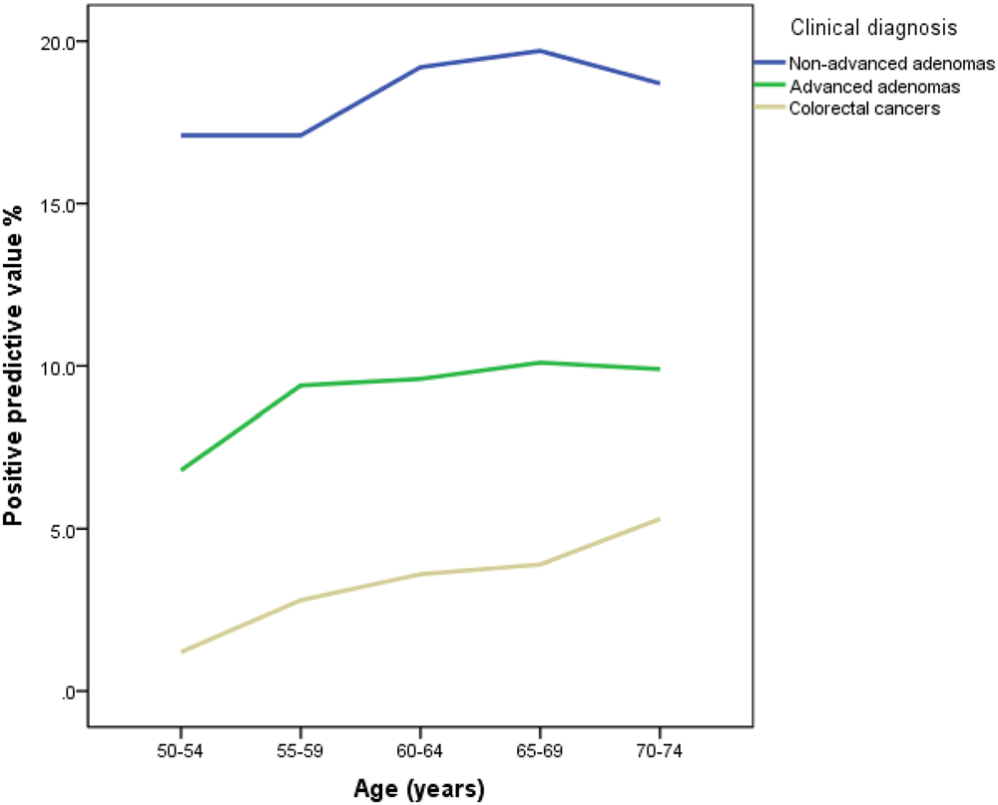
Positive predictive values (PPVs) of colorectal cancers, advanced adenomas and non-advanced adenomas by age.

In 10,600 colonoscopies, 351 (3.3%, 95% CI: 3.0–3.7) CRCs, 980 (9.2%, 95% CI: 8.7–9.8) AAs, and 1,961 (18.5%, 95% CI: 15.9–18.3) non-advanced adenomas were detected (Table 2). All CRCs and AAs were verified by histopathology. The PPVs of CRCs in the exclusively FIT-positive population and population positive on both the FIT and the HRFQ were 4.9% and 5.3%, respectively, which were 10 times greater than the PPV in the exclusively HRFQ-positive population (0.5%). The PPVs of AAs in the exclusively FIT-positive population and the population positive on both tests were 2 times greater than the PPV in the exclusively HRFQ-positive population, but the PPVs of non-advanced adenomas (NAA) were similar among the three groups. Male subjects were found to have more CRCs and AAs than female subjects (4.5% VS 2.5% and 11.9 VS 7.3%, respectively). There were similar PPVs for cancers in subjects from urban and rural areas (3.2% VS 3.6%), but the PPVs of AAs were higher in subjects from rural areas than in those from urban areas (11.4%, 95% CI: 10.2–12.6 VS 8.5%, 95% CI: 7.9–9.2). Figure 2 shows that the PPVs of CRCs, AAs, and non-advanced adenomas increased with increasing age, except for non-advanced adenomas in the 70–74 year group. The curves for cancer increased sharply, while the curve for AAs became smooth from 55 to 74 years old.

**Table 2:** Positive predictive values (PPV) among 10,600 colonoscopies.

	Colonoscopies performed n	NAA % (95% CI)	AA % (95% CI)	CRC % (95% CI)	χ^2^ test
**Total**	10600	18.5 (17.8–19.2)	9.2 (8.7–9.8)	3.3 (3.0–3.7)	
**Subgroup**					
HRFQ+	4007	17.1 (15.9–18.3)	5.0 (4.3–5.7)	0.5 (0.4–0.6)	χ^2^ = 365.761 P < 0.001
FIT+	5211	20.1 (19.0–21.2)	12.1 (11.2–13.0)	4.9 (4.3–5.5)	
Both+	1382	16.6 (14.6–18.7)	10.7 (9.1–12.3)	5.3 (4.1–6.5)	
**Age**, **years**					
50–54	1835	17.1 (15.4–18.8)	6.7 (5.6–7.9)	1.2 (0.7–1.8)	χ^2^ = 80.963 P < 0.001
55–59	1980	17.1 (15.7–18.8)	9.4 (8.1–10.7)	2.8 (2.1–3.6)	
60–64	2988	19.2 (17.8–20.6)	9.6 (8.6–10.7)	3.6 (3.0–4.3)	
65–69	2719	19.7 (18.2–21.2)	10.1 (9.0–11.2)	3.9 (3.2–4.7)	
70–74	1078	18.7 (16.4–21.0)	9.9 (8.1–11.7)	5.3 (4.0–6.6)	
**Sex**					
Male	4480	22.1 (20.9–23.3)	11.9 (11.0–12.9)	4.5 (3.9–5.1)	χ^2^ = 207.751 P < 0.001
Female	6120	15.9 (15.0–16.8)	7.3 (6.6–7.9)	2.5 (2.1–2.8)	
**Residence**					
Urban	8004	17.2 (16.4–18.0)	8.5 (7.9–9.2)	3.2 (2.8–3.6)	χ^2^ = 72.900 P < 0.001
Rural	2596	22.7 (21.1–24.3)	11.4 (10.2–12.6)	3.6 (2.9–4.3)	

Advanced adenoma was defined as an adenoma with a diameter of 10 mm and/or with a 25% villous component and/or high grade dysplasia. HRFQ: high-risk factor questionnaire, FIT: faecal immunochemical test, NAA: non-advanced adenoma, AA: advanced adenoma, CRC: colorectal cancer.

### Colorectal cancer stages

Among the 351 CRCs found by screening, 4 patients could not be staged. Two patients declined further medical examination and operation after colonoscopies for individual reasons. Another 2 patients refused to provide information about their treatment after the diagnosis of cancer, and their stages were omitted in the data reported by hospitals to the Guangzhou Registry. The detection ratio of localized CRCs (including stage I and stage II) was 67.9% (235/351). Comparably, the contemporaneous detection ratio of localized CRCs from the registration system report was only 40.4% (1,152/2,852). Therefore, the detection ratio of localized CRCs increased 68.1% due to screening, including a 114.4% increase in patients with stage I CRC (Table 3). Upon further analysis of the subgroups, the detection ratio of localized cancers in the exclusively HRFQ-positive population was lower than that in the exclusively FIT-positive population or in the population positive on both tests (61.9% vs 67.9%, 67.1%, respectively).

**Table 3:** AJCC stages of colorectal cancers from screening and report.

Age, years	Unknown stage n (%)	AJCC stages			
		I, n (%)	II, n (%)	III, n (%)	IV, n (%)
**System report**					
50–54	42 (10.1)	76 (18.3)	86 (20.7)	110 (26.5)	101 (24.3)
55–59	31 (6.5)	72 (15.1)	103 (21.6)	137 (28.8)	133 (27.9)
60–64	62 (8.7)	114 (15.9)	176 (24.6)	182 (25.5)	181 (25.3)
65–69	65 (9.3)	132 (18.9)	164 (23.4)	175 (25.0)	164 (23.4)
70–74	57 (10.5)	103 (18.9)	126 (23.1)	133 (24.4)	126 (23.1)
Total	257 (9.0)	497 (17.4)	655 (23.0)	737 (25.9)	705 (24.7)
**Screening**					
50–54	1 (4.5)	8 (36.4)	7 (31.8)	4 (18.2)	2 (9.1)
55–59	0 (0.0)	14 (25.0)	16 (28.6)	21 (37.5)	5 (8.9)
60–64	0 (0.0)	40 (36.7)	32 (29.4)	24 (11.9)	13 (11.9)
65–69	1 (0.9)	43 (40.2)	32 (29.9)	18 (16.8)	13 (12.1)
70–74	2 (3.5)	26 (45.6)	17 (29.8)	10 (17.5)	2 (3.5)
Total	4 (1.1)	131 (37.3)	104 (29.6)	77 (21.9)	35 (10.0)
**Subgroup**					
HRFQ+	0 (0.0)	7 (33.3)	6 (28.6)	5 (23.8)	3 (14.3)
FIT+	2 (0.8)	98 (38.7	75 (29.2)	58 (22.6)	24 (9.3)
Both+	2 (2.7)	26 (35.6)	23 (31.5)	14 (19.2)	8 (11.0)

AJCC: American Joint Committee on Cancer.

## Discussion

This is a community-based colorectal cancer screening study assessing the success of mass mobilization in a population of over 2 million subjects. The data provide an interesting comparator for countries considering or planning the implementation of population-based screening. We observed low attendance rates for primary screening and colonoscopies but high PPVs for AAs and CRCs. Increasing numbers of countries are encouraging nationwide CRC screening programmes. Several easy and efficient screening strategies for colorectal neoplasms are now available^14^. Screening strategies should be chosen on the basis of individual risk, personal preference, and access. Both HRFQs and FITs were used as primary mass screening methods, and colonoscopy was used as a secondary screening method in China. The China National Committee of Cancer Early Detection and Treatment proposed the revised screening protocol in 2006. Meng *et al*.^16^ reported that although CRC detection rates were not improved by HRFQ, it could be used as a complementary primary screening method for colorectal adenomas and non-adenomatous polyps to compensate for a deficiency in FITs. In our study, HRFQs found nearly as many NAAs as FITs but far fewer AAs and CRCs. To date, FITs are still one of the most efficient screening methods for identifying CRC cases without obvious risks and complications^14^, but FITs overlook AAs and NAAs without bleeding, which may be discovered by the HRFQ^24^. A study in Hong Kong demonstrated that a risk scoring system, similar to the HRFQ in this study, was useful^9^. A recent meta-analysis reported that both sigmoidoscopy and colonoscopy prevent the majority of deaths from colorectal cancers^25^. Nevertheless, colonoscopy is relatively invasive, labour intensive, and expensive. Colonoscopy also requires a high level of expertise, so it might not be appropriate as a first-line test in regions with fewer resources or for the first stage of screening to identify individuals at high risk of cancer/advanced adenomas from among the average-risk population. The HRFQ is simple and easy to administer. The HRFQ takes approximately 5 minutes to complete by self-administration or by an investigator. In our study, all attendants finished the HRFQs, while only 91.0% of attendants finished the FITs.

A very important early indicator of an effective population-based screening programme is uptake. Obviously, participation depends on the willingness of participants^26^. Uptake in Europe and North America is high, where uptake for FOBT or FIT studies has varied from 40% to 70%^27^. In China, a meta-analysis shows that the compliance rates for a questionnaire survey, FOBT and colonoscopy are 56%, 50% and 44%, respectively^28^. Our study had relatively low attendance rates for both the HRFQ (15.4%) and FIT (14.0%). We invited residents to the first stage of screening by public media (newspaper and TV) and reminders from a mobile SMS instead of house visits or telephone calls. Initially, with this model of mass mobilization, the GP cannot communicate with participants face-to-face or by voice. Although this model of mass mobilization takes less time, the involvement of GPs in colorectal cancer screening can be very effective in enhancing compliance^29^. A meta-analysis concluded that the attendance rate was usually low because of a lack of knowledge of cancer and screening, screening costs, a feeling of embarrassment, a fear of screening complications or discomfort, barriers to the implementation of screening, a lack of communication with physicians and a lack of symptoms and awareness^30^. Two recent mass screenings in China in which the same methods were employed reported similar results to ours, including the low rates of colonoscopy attendance in the population identified as positive in the initial screenings^18,20^.

During the 3 years of screening, the attendance rate for colonoscopies was 18.9%, which was relatively low compared to most randomized controlled trials. The first stage of screening was free for all participants and financially supported by the Guangzhou government. The second stage of screening with colonoscopy had to be paid for by the participants themselves. Even in wealthy countries, a lack of health insurance and dual coverage with Medicare and Medicaid were the most frequently reported barriers, whereas Medicare’s coverage of screening colonoscopy was consistently reported as a facilitator^31^. Payment by the patient may be another reason for the low attendance rate for colonoscopies in our study. Mass screening in large-scale populations usually results in low attendance rates for colonoscopy. A nationwide study in Germany showed that the cumulative participation rate was 17.2% of eligible women and 15.5% of eligible men 55 to 74 years old from January 2003 to December 2008^10^, which is in line with our results.

Our attendance rates increased with increasing age in the first stage of screening but decreased in the second stage of screening. First, most of the relatively young population (<60 years old) is still at work. This population does not have as much free time as elderly people who have retired. Second, the younger generation does not perceive the necessity for CRC screening. As demonstrated by other researchers, these are the major obstacles to participation in a mass screening programme^26,32,33^. In most studies, the main barrier to screening was a poor understanding of the goals of screening and the perception that screening was necessary only when symptoms had developed. The young participants had lower attendance rates and positive rates in the first stage of screening, while they contributed more to the colonoscopy uptake. Perhaps this is because the young participants thought they were “normal” but when the HRFQs or FITs were positive, they believed they might be at risk for CRC and were interested in understanding their true status.

Most studies reported that male subjects had higher attendance rates for FOBT or FIT, especially in Europe and North America^27^. However, we found a higher attendance rate in the first stage of screening among female subjects rather than among male subjects (19.2% vs 11.5%), with female subjects having almost double the rate of male subjects. The reason may be that female subjects traditionally have a stronger awareness of health and compliance with authority compared to male subjects; unfortunately, the positive rates on HRFQs and FITs and the PPVs for cancers are higher in male subjects. The incidence rate of CRCs in male subjects is almost double that in female subjects^5^, which suggests that extra effort should be devoted to the mobilization of the male population in future screenings. In the following round of screening, the male and senior populations should be paid extra attention to raise their attendance rates for colonoscopies.

Overall, the PPV was 3.3% for CRCs, and age-specific PPVs increased with increasing age from 1.2% in the 50- to 54-year-old group to 5.3% in the 70- to 74-year-old group. The attendance rates for colonoscopy in the positive population decreased with increasing age, whereas age-specific PPVs for AAs simultaneously increased with increasing age. These observations suggest that elderly people are more apt to complete their colonoscopies. Reports on colorectal cancer screening in populations of millions of people are limited. France performed a nationwide screening programme. Pallet *et al*.^34^ reported 23% of 620,227 eligible Parisians underwent the FITs, with 4.3% positivity and 733 (30.5%) AAs and 205 (8.5%) CRCs identified among the 2,401 colonoscopies from 2016 to 2017. The lower positivity rate for FITs and higher PPVs for AAs and CRCs observed in that study are related to the higher cut-off value chosen for the FITs. The cut-off value for FITs was 30 μg Hb/g faeces in France, whereas the cut-off value for FITs in Guangzhou was 20 μg Hb/g faeces.

One of two main aims of CRC screening is to detect asymptomatic early-stage cancers. In our study, invasive cancers were found in 351 patients, with the majority of diagnosed cancers being localized (AJCC I/II) (67.9%). This finding is in line with data from another screening colonoscopy study^10^. There was a 68.1% increase in localized CRCs (AJCC I/II) compared to the contemporaneous local system report (40.4%). The detection ratio increase for stage I CRC by screening reached 114.4% (from 17.4% to 37.3%) (Table 3). Another main aim of colonoscopy screening is to detect as many AAs as possible and remove them. A study with 840,149 screening colonoscopies confirmed the higher risk of these lesions to progress to cancer with a 10-year risk of approximately 25% for a 55-year-old^35^. Nine hundred eighty (9.2%) advanced adenomas among 10,600 colonoscopies were found and removed in our study. Therefore, this round of screening in Guangzhou was significant not only for early detection but also for the prevention of CRCs.

This study has several limitations. This study is the first round of colorectal cancer screening we have implemented citywide with over 2 million in the target population. First, we invited residents to the first stage of screening by public media (newspaper and TV) and reminders from a mobile short message service instead of by house visits or telephone calls. Many target residents may not have noticed the information or have received electronic invitation letters, which meant that the eligible population was overestimated, and low uptake rates were reported. Second, the attendance rates for colonoscopy were low in our study. Further efforts, including free colonoscopies and raising resident awareness of the risks, should emphasize the need for 80% of positive participants to complete colonoscopies. Finally, selection bias may exist in this mass screening. People who experienced chronic stomach discomfort (e.g., coprostasis, diarrhoea and bloody faeces) or had a first-degree relative with CRC were more inclined to take part in the screening, which may result in higher positive rates of FITs/HRFQs and PPVs of colonoscopies that readers should interpret cautiously. If the targeted population was informed by the contractual GPs in the next round of screening, the bias in the selection of participants would be improved. In this mass population-based CRC screening study, the uptake rates were low, including the target population attending primary screening in the first stage and the positive participants attending colonoscopy in the second stage. The PPVs for CRCs, AAs and non-advanced adenomas were high. More effective communication regarding the programme and subsidies should be taken into account for colonoscopies to increase compliance. Although the HRFQ is more convenient and generally more accepted than the FIT, it is less efficient at detecting CRCs and AAs. Further research is needed to confirm the value of the HRFQ in the field.
